# Biochemical and reproductive biomarker analysis to study the consequences of heavy metal burden on health profile of male brick kiln workers

**DOI:** 10.1038/s41598-022-11304-7

**Published:** 2022-05-03

**Authors:** Mehwish David, Sarwat Jahan, Javaid Hussain, Humaira Rehman, Karen J. Cloete, Tayyaba Afsar, Ali Almajwal, Nawaf W. Alruwaili, Suhail Razak

**Affiliations:** 1 Reproductive Physiology Laboratory, Department of Zoology, Quaid-I-Azam University, Islamabad, Pakistan; 2 National Centre for Physics, Quaid-I-Azam University Campus, Islamabad, 44000 Pakistan; 3 Biomedical Center, Uppsala University, Husargatan 3, 752 37 Uppsala, Sweden; 4 College of Graduate Studies, UNESCO-UNISA Africa Chair in Nanosciences-Nanotechnology, University of South Africa, Muckleneuk Ridge, PO Box 392, Pretoria, South Africa; 5 Nanoscience’s African Network (NANOAFNET), iThemba LABS-National Research Foundation, 1 Old Faure RoadWestern Cape Province, PO Box 722, Somerset West, 7129 South Africa; 6 Department of Community Health Sciences, College of Applied Medical Sciences, King Saud University, Riyadh, Saudi Arabia

**Keywords:** Zoology, Diseases

## Abstract

The present study aims to assess the effect of a heavy metal burden on general health, biochemical parameters, an antioxidant enzyme, and reproductive hormone parameters in adult male brick kiln workers from Pakistan. The study participants (*n* = 546) provided demographic data including general health as well as body mass index. Blood was collected to quantitatively assess hematological, biochemical, and reproductive hormone parameters as well as heavy metal concentrations using both atomic absorption spectroscopy (AAS) and particle-induced X-ray emission (PIXE). The data showed that 10% of the brick kiln workers were underweight and 10% obese (*P* = 0.059), with workers also reporting multiple health issues. Heavy metal concentrations utilizing AAS revealed significantly (*p* = 0.000) higher levels of cadmium, chromium, and nickel, while PIXE detected more than permissible levels of Si, P, S, Cl, K, Ca, Zn, Ti (*p* = 0.052), Mn (*p* = 0.017), Fe (*p* = 0.055), Co (*p* = 0.011), Ni (*p* = 0.045), and Cu (*p* = 0.003), in the blood of kiln workers. Moreover, a significant increase in platelet count (*P* = 0.010), a decrease in sodium dismutase levels (*p* = 0.006), a major increase in reactive oxygen species (*p* = 0.001), and a reduction in protein content (*p* = 0.013) were evident. A significant increase in cortisol levels (*p* = 0.000) among the workers group was also observed. The concentration of LH and FSH increased significantly (*p* = 0.000), while that of testosterone decreased (*p* = 0.000) in the worker group compared with controls. A significant inverse relationship was found between cortisol, LH (r =  − 0.380), and FSH (r =  − 0.946), while a positive correlation between cortisol and testosterone was also evident (r = 0.164). The study concludes that increased heavy metal burden in the blood of brick kiln workers exposes them to the development of general and reproductive health problems due to compromised antioxidant enzyme levels, increased oxidative stress conditions, and a disturbing reproductive axis.

## Introduction

Pakistan is the third largest brick producing country in South Asia, with more than 45 billion bricks being produced per year by approximately 1.8 million brick kiln workers^1,2^. A recent study reported that five million people are known to be associated with the brick industry^3^. The Punjab province of Pakistan contains the largest number (10,347) of active brick kilns that are operated by approximately 249 682 brick kiln workers^4^. Despite the large numbers of families associated with this profession, it remains a highly unstructured and undocumented area in terms of public and reproductive health risks^5^.

Numerous studies have shown that brick kiln workers are exposed to brick kiln pollutants including heavy metals that are released during the combustion of low quality fuels such as rubber tires and motor oils as well as from other sources such as brick kiln soil^6^. Heavy metals impart hazardous effects on public and reproductive health of brick kiln workers^7,8^. More specifically, different study groups have reported that exposure to elevated levels of heavy metals such as lead, cadmium, chromium, and other pollutants in the brick kiln environment induces neurological, gastrointestinal, and pulmonological pathologies and even neoplasms^9–11^. Furthermore, multiple hematological and immunological pathologies, targeting heme synthesis enzymes, thiol-containing antioxidants and other enzymes have also been reported^9,10,12^. Some of the heavy metals found in biological systems such as cadmium, chromium and nickel are non-essential for human health, however, their toxic actions have been reported^13–15^. Cadmium is accumulated in liver and kidney tissues of human and acute Cd toxicity may cause musculoskeletal and cardiovascular diseases^15^. Cadmium exposure results in hemorrhage, edema, cell death and tubular destruction. Its half-life in human blood is of 2–3 months, however, following absorption it can reside in body tissues for years^16^. Cd and Chromium are carcinogenic in nature and are known to act as an endocrine disruptor^17,18^. Chronic exposure of Cr to human body imparts hazardous effects on liver, kidney and lungs and is highly toxic to biota^17^. Occupational exposure to Cr and its compounds may affect 300,000 workers annually and is a major concern for causing Cr-related diseases in industrial workers^19^. Prolonged Ni and Cr poisoning may induce pulmonary and nervous diseases, as well as may affect the cell cycle by inducing lungs cancer and cellular impairments in liver and heart^13,14^. Zinc acts as a functional and structural element for human health and plays an important role in cancer etiology and human growth^15^. Heavy metals are also reported to induce reproductive toxicity in both males and females^20–23^.

Besides heavy metal exposure, occupational factors within the brick kiln environment including job length, work type, and fuels used including the lack of protective equipment, have an additional deleterious impact on brick kiln worker health^24,25^. For example, a recent study conducted in Mexico reported a direct correlation between job duration and severity of pulmonary disorders among brick kiln workers^26^. In Pakistan, various researchers have conducted studies that have focused on occupational factors of brick kiln workers related to demographic and health-related variables. For example, a previous study conducted by Shaikh et al. in the Sindh province of Pakistan (Larkana and Dadu) addressed the prevalence of respiratory diseases and their association with work type including brick carriage and placement, brick molding, and brick baking process, for male brick kiln workers among whom these tasks are more prevalent^25^. Studies performed by Muhammad et al. at Badhber, Peshawar, further reported that most of the brick kilns in Pakistan employs traditional methods of brick manufacturing linked to hard physical labor stretching well beyond the work duration time approved by international law^27^. For brick kiln workers in Pakistan, illiteracy, the unavailability of alternative income sources, and poor socio-economic circumstances, adds an additional layer to occupational factors responsible for poor health outcomes. In community members with poor health outcomes, continuous exposure to brick kiln pollutants further exacerbated by occupational factors may add to an already worrying burden of disease among such communities^28^. In sum and based on the morbidity risks of exposure to brick kiln pollutants accompanied by various occupational factors, human biomonitoring studies are important^29^.

Human biomonitoring involves screening of biomarkers such as body fluids including blood, urine, saliva, breast milk, sweat, as well as hard tissues such as hair, teeth, and nails^30^. Of these tissues, blood is one of the most popular biomatrices screened for metal exposure in HBM surveys since it is in contact with and in equilibrium with body organs and tissues exposed to metals^31^, whilst the sampling procedures for blood have also been standardized. Different techniques have been established to quantitatively screen biomatrices for metals and trace elements. Popular methods include atomic absorption spectroscopy (AAS), inductively coupled plasma–mass spectrometry, and isotopic fingerprinting for screening metals^32^. These techniques however require laborious, time consuming, and destructive sample preparation that may introduce contaminants into the sampled tissue. In recent decades, a technique known as proton induced X-ray emission (PIXE) has been introduced that can quantitatively detect metals and trace elements in human blood with minimal sample preparation^33,34^. Proton induced X-ray emission has been described as one of the most promising and versatile non-destructive elemental screening techniques^35^ based on its ability to simultaneously quantify several elements heavier than Na at μg/g-level sensitivity in small sample sizes that require minimal processing^36^. Often, PIXE have been used as a complementary technique to AAS analysis to provide the full quantitative profile of elements in a sample^37^.

To date, few studies in Pakistan have been designed to monitor heavy metal exposure, health outcomes including biochemical and reproductive parameters, occupational factors, and socio-demographic variables among male workers exposed to a brick kiln environment. Such studies are not only important to add to the body of literature on brick kiln occupational health risks, but also to build an evidence base to advise the fast-tracked implementation of safety regulations within the brick kiln industry. Jahan et al. (2016) conducted a study at Punjab (Rawat) for which male workers were recruited from Tarlai, District Rawalindi, and their biochemical profile evaluated to determine the effect of brick kiln emissions on reprodutive health. The study reported that heavy metal burden in blood may cause oxidative stress, resulting in reproductive dysfunctions by inducing hormonal impairments. However, this study screened a different area and population (Tarlai), whilst the current study focuses on yet another population (Gujar Khan region), adding screening of cortisol as a stress hormone known to affect reproductive parameters, the use of benchtop elemental analysis techniques in combination with an ion beam elemental analytical technique, PIXE, to ascertain the full complement of metals and other elements in blood samples of workers, and finally, different statistical models to approach data analysis. Specific objectives of the study included (1) to compare the socio-demographic data of workers versus control samples residing outside of the immediate brick kiln area; (2) to compare elemental and metal content, blood parameters, antioxidant enzyme profile, cortisol, and reproductive hormone levels in blood of workers versus controls; and (3) to use multivariate statistics to compare associations between hormone status and years of living at the brick kiln site, cortisol and reproductive hormone levels, as well as between group data.

## Materials and methods

### Study design and ethical clearance

The study was conducted at the Reproductive Physiology Laboratory, Department of Zoology, Faculty of Biological Sciences, Quaid-i-Azam University in Islamabad, Pakistan. Blood sampling were completed from March 2018 to November 2018 at different brick kiln sites in Rawat (District Rawalpindi, Punjab). The area map has been provided in a previous report^20^. The approval to conduct this study was obtained by the Bio-Ethical Committee of the Department of Zoology, Quaid-i-Azam University, Islamabad and was assigned protocol # BEC-FBS-QAU2018-97. Participants of this study also signed informed consent forms.

### Study area

A survey was conducted and different brick kiln sites from District Rawalpindi (Potohar) were selected^20^. District Rawalpindi contains 238 functional brick kilns of which 108 brick kilns are in the Gujar Khan region near Rawat, Islamabad, that was selected on the basis of production capacity, functionality, nearness to communities and operation throughout the year. Approximately 50 brick kilns were visited out of which 25 brick kiln owners agreed to participate in the study. All the brick kilns were similar in that either they were Fixed Chimney Bull’s Trench Kilns or used zigzag technology for making bricks, whilst the number of employees varied based on ethnicity and gender. In certain kilns, only male workers were dominant, whilst others had women and children or only Pathan/Punjabi workers. The rate of participation was quite low and varied among different kilns due to multiple reasons, i.e. certain brick kilns were not registered and therefore their owners did not agree to participate; prevalence of bonded and child labor; and illiteracy as well as the occurrence of superstitious beliefs. More detailed information on the study area can be found in our previous work^20^.

### Study population

Adult males employed for a varied number of years at the brick kiln sites were selected for this study. Random selection was done for participants willing to be included in this study, with everyone representing a population. After data collection, the data was sorted, analyzed and subsequently only those participants included who fulfilled our study objectives. The sample size was calculated using Eq. (1) as previously described^24^ which provided a calculated sample size of 336. Because the study focused on the health status of individuals, the sample size was calculated with 5% level of significance and 10% minimum error based on the number of healthy and diseased workers.

Equation (1):

1 $$n = {\text{4pq}}/{\text{L2}}$$

where *n* = Sample size, *P* = Workers who had some health problems, q = Workers who had no health problems, L = Level of significance.

**Figure 1:**
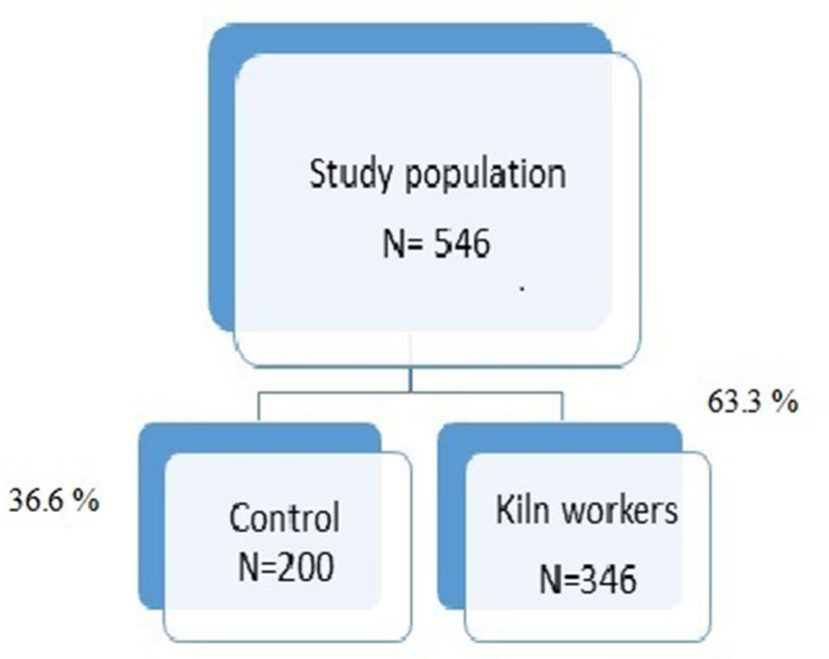
Flowchart of the study population.

The final total number of n = 346 adult male workers aged 18–55 years and working for at least 1–5 years and more (< 1yrs- > 20yrs) at brick kiln sites were included in the study. Control samples included adult males (aged 18–58 years; *n* = 200) who lived in the same district, but at least 40 km away from the kilns. Control participants’ least exposed to environmental pollutants such as vehicle and industrial smoke were selected. The flowchart of the study population is provided in Fig. 1.

### Data collection

#### Interviews

The participants were interviewed with a structured, validated questionnaire to obtain information regarding their socio-demographic factors such as age, weight, height, marital status, education status, monthly income, health and disease history, sleeping duration, family history of brick kiln work, living history in the area, type of work at the brick kiln sites, and smoking habits. Health and disease history focused on diseases associated with exposure to brick kiln environments as well as headache, body, and leg pain as other types of health concerns. Based on the information obtained, male participants were classified into the following two groups: Group I, Control (living in the same area) and Group II, Brick-kiln workers. All samples were anonymized before being processed.

#### Body mass index

Body mass index for male participants was assessed using a previously described method^38^.

#### Blood sample collection

Blood samples from workers and control participants were collected by venipuncture in a 5 ml syringe and divided into two halves that were placed in either EDTA coated tubes or glass tubes. Blood contained in the EDTA tubes was used for complete blood count and heavy metal determination with AAS and PIXE analysis. Blood in the glass tubes was centrifuged at 13,000 revolutions per minute for 10 min and the plasma collected and stored at − 20 °C until further analysis. The study protocol was carried out in accordance to the principles of the Declaration of Helsinki^43^.

#### Heavy metal determination

##### Atomic absorption spectroscopy

For heavy metal determination, acid digestion for blood samples was performed following a previously described protocol and the processed samples were analysed using a Fast Sequential Atomic Absorption Spectrometer (Varian, AA240FS, USA)^39^. The instrument was calibrated by using multiple concentrations from 1–50 ppm and calibrations were done for each metal using the formula (C1V1 = C2V2), and a series of standards run to deliver a calibration curve.

##### External beam PIXE analysis: Preparation of blood samples for PIXE analysis

Refrigerated blood samples were thawed and kept at room temperature before preparation for PIXE analysis. About 20–30 µl of blood was pipetted onto 99.99% pure copper strips coated with carbon tape. The blood films were air-dried for about 30–40 min at room temperature (25 °C) and 40% humidity. All samples were handled with latex gloves to avoid contamination.

##### PIXE procedure

To determine the metal profile and levels in blood samples, the nuclear microprobe at the National Centre for Physics, Pakistan was used. A proton beam of 3 MeV energy was provided by a 5 MV Pelletron Tandem Accelerator and a previously described protocol used^40^. Samples were placed in a vacuum chamber at a pressure of 10^–7^ torr and irradiated with the proton beam. All samples were measured with an ion current of 3^–5^ nA and an integrated beam charge of 2 μC. Emitted X-rays from blood samples were detected using a Sirius SD detector with a resolution of 129 eV positioned at an angle of 45° with respect to the incident beam. A Mylar absorbing foil of 40 μm thickness was placed in front of the detector to minimize the intensity of low energy X-rays emitted from the matrix elements. Spectral fitting was performed with GUPIXWIN software (shown in supplementary Fig. 1)^40^. PIXE analysis of worker and control blood samples produced data with an accuracy of 15% ± 5% within the standard tolerance limit. For better absolute results, the detector was calibrated with K-shell X-rays of a 99.99% pure Copper standard of 20 µm thickness and a TiV standard (NIST) before analyzing the blood samples (supplementary Fig. 2). The system constant or H-values, including all electronics and system errors were corrected to 0.001531 sr. Using the standard reference material Bovine Lever SRM 1577c, the reproducibility of our PIXE analysis system was of the order of < 5.5% or better.

#### Complete blood count measurement

Collected blood was kept in a lavender vacutainer containing EDTA K_3._ Blood count and complete blood picture was performed using an automated hematology analyzer Sysmex KX21, Meditechat Multilink Laboratory, Rawalpindi^41^.

#### Biochemical studies

Serum samples were analyzed for antioxidant enzyme activity of SOD (units/mg of protein), POD, and lipid peroxidation via Malondialdehyde (MDA) on a UV Spectrophotometer (Agilent 8453), while oxidant concentrations of reactive oxygen species (ROS) was determined using previously established methods^42–45^. The systems were calibrated with standards and comparison with reported literature was made. Serum total protein content was quantitatively determined using a commercially available kit from AMEDA (Labordiagonstik GmbH Krenngasse, Graz/Austria)^20^.

#### Enzyme-linked immunosorbent assay

Hormonal concentrations in blood serum were determined by different commercially available enzyme immune assay tests kits for luteinizing hormone (Reddot biotech INC), follicle-stimulating hormone (Reddot biotech INC), testosterone (Amgenix Inc, USA), and cortisol (The Calbiotech, Inc. USA). All the assays were performed following the protocols provided with the kit. Positive and negative controls were also run alongside standards for quality control of assays.

#### Statistical analysis

The data is represented as the mean and standard error of the mean (Mean ± SEM). Descriptive statistics of socio-demographic variables were computed as mean, standard deviation, and percentage. The p values were calculated using a one-sample t-test to compare socio-demographic data between test and control participants. An unpaired student t-test in SPSS 17 were used to compare test and control participants in terms of heavy metal burden, blood parameters, antioxidant enzymes profile, and hormonal concentrations. Pairwise Pearson’s correlation was used to assess the correlation between hormones and the number of years of living at the brick kiln sites as well as between cortisol and reproductive hormones levels.

#### Principal component analysis

Principle component analysis was performed using PAST (Version 3.14) to determine relationships between the collected data for both groups. Significance was set at *p* < 0.05.

### Ethics approval and consent to participate

The approval to conduct this study was obtained by the Bio-Ethical Committee (BEC) of the Department of Animal Sciences, Quaid-i-Azam University, Islamabad and was assigned protocol # BEC-FBS-QAU2018-97. Participants of this study signed informed consent forms before collection of data and blood samples, with a promise that their identity will be kept anonymous.

## Results

### Sociodemographic data

A total of 546 men were examined for the study, of which test participants (*n* = 346) had a mean ± SEM age of 23.0 ± 0.23 and control participants (*n* = 200) had a mean age of 26.9 ± 0.77 years. Of the workers, 18% were overweight, 10% obese, and 10% underweight, whilst 59.5% had a normal body weight (Table 1). Individuals exposed to brick kiln pollutants were experiencing various health issues including stomach problems (10%), asthma (8%), kidney disorders (5%), skin allergies (1%), tuberculosis (3%), and other mild symptoms of flue, cold, and headache (38%). Regular medication (Panadol and Paracetamol) for headache or stomach issues was ascribed to 21% of workers. The majority of workers (58.3%) were addicted and consuming tobacco in various forms such as Cigarette, Naswaar (an amalgamation of tobacco leaves, calcium oxide and wood ash), Charras (hashish form of cannabis), and Hukka (tobacco mixture). 77% of workers were married, 71% had a sleep duration between 8 and 12 h, and 84% were illiterate. Participants were working at very low wages ranging from PKR 10,000–20,000 per month (Table 2), with significantly (*p* = 0.002) more of the workers (*n* = 178, 51%) earning PKR 10,000–15,000 than control participants (*n* = 18, 9%). Mean years of living at the brick kiln site was 14 years, the mean job duration 12 years, and mean exposure time to kiln emissions was 10 h per day. None of the participants were wearing protective gear, e.g., gloves, head covers, respirators, or eye protection.

**Table 1:** Sociodemographic characteristics of surveyed brick kiln male workers from Rawalpindi, Pakistan.

Socio-demographic parameters					*P* value statistics
Parameters	Control		Workers		
Age (years)	*n*	Percentage	*n*	Percentage	
18–28	114	57.0	186	53.7	*P* = 0.009
29–38	32	16.0	66	19.0	*P* = 0.001
39–45	36	18.0	34	9.83	*P* = 0.000
45 +	18	9.00	60	17.3	*P* = 0.096
**BMI (kg/m**^**2**^**)**					
Normal weight	42	21.0	206	59.5	*P* = 0.701
Obesity	68	34.0	38	10.9	*P* = 0.059
Overweight	78	39.0	64	18.5	*P* = 0.174
Underweight	12	6.00	38	10.9	*P* = 0.865
**Marital status**					
Married	98	48.4	268	77.4	*P* = 0.277
Unmarried	102	51.5	78	22.5	*P* = 0.084
**Sleep duration (hours)**					
less than 8	56	28.0	96	27.7	*P* = 0.045
8–12	126	63.0	248	71.6	*P* = 0.465
12 +	18	9.00	2	0.58	*P* = 0.000
**Education status**					
Illiterate	2	1.00	292	84.3	*P* = 0.496
Primary	0	0.00	40	11.5	*P* = 0.500
Matric	54	27.0	6	1.73	*P* = 0.033
Intermediate	60	30.0	8	2.31	*P* = 0.416
Bachelors	36	180	0	0.00	*P* = 0.500
Masters	36	18.0	0	0.00	*P* = 0.500
Higher studies	12	6.00	0	0.00	*P* = 0.500
**Health history**					
Allergy	6	3.00	6	1.73	*P* = 1.00
Asthma	0	0.000	28	8.09	*P* = 0.500
Diabetes	6	3.00	2	0.58	*P* = 0.295
Obesity	0	0.000	2	0.58	*P* = 0.500
Stomach issue	0	0.000	36	10.4	*P* = 0.500
Hyperandrogenism	0	0.000	0	0.00	*P* = 1
Kidney issue	0	0.000	18	5.20	*P* = 0.500
Tuberculosis (TB)	8	4.00	12	3.47	*P* = 0.500
Hepatitis	0	0.000	6	1.73	*P* = 0.126
Other	30	15.0	132	38.1	*P* = 0.358
None	150	75.0	104	30.0	*P* = 0.114
**Medication**					
Yes	14	7.00	76	21.9	*P* = 0.381
No	42	21.0	174	50.2	*P* = 0.349
No need	144	72.0	96	27.7	*P* = 0.126
**Smoking status**					
Smokers	202	58.3	44	22	*P* = 0.363
Non smokers	144	41.6	156	78	*P* = 0.025

**Table 2:** Table showing work history of men working at brick kilns at Rawalpindi, Pakistan.

Work history					*P* value statistics
Parameters	Control		Workers		
Income (PKR/month)	*n*	Percentage	*n*	Percentage	
< 10,000	74	36.4	52	15.0	*P* = 0.000
10,000–15,000	18	9.09	178	51.5	*P* = 0.002
16,000–20,000	18	9.09	98	28.3	*P* = 0.000
20,000 +	90	45.5	18	5.20	*P* = 0.000
**Work type**					
Carriage and placement	0	0.000	88	25.4	*P* = 0.500
Bakers	0	0.000	78	22.5	*P* = 0.500
Molders	0	0.000	124	35.8	*P* = 0.500
Non-workers	26	13.0	8	2.31	*P* = 0.310
Others	174	87.0	48	13.8	*P* = 0.329
**Years of living** **(years)**					
< 1	0	0.00	94	27.1	*P* = 0.500
1–5	68	34.0	96	27.7	*P* = 0.035
6–10	30	15.0	52	15.0	*P* = 0.000
11–20	24	12.0	56	16.1	*P* = 0.000
20 +	78	39.0	48	13.8	*P* = 0.002
**Work duration (years)**					
1–5	NA	NA	56	16.1	*P* = 0.003
6–10	NA	NA	72	20.8	*P* = 0.000
11–20	NA	NA	108	31.2	*P* = 0.814
20 +	NA	NA	110	31.8	*P* = 0.446
**Exposure time (hr/day)**					
< 1	0	0.000	4	1.2	*P* = 0.016
1–5	104	52.0	24	6.9	*P* = 0.000
6–10	66	33.0	132	38.1	*P* = 0.168
11–15	30	15.0	178	51.4	*P* = 0.000
15 +	0	0.000	8	2.31	*P* = 0.002

### Element determination

#### External beam PIXE analysis

**Figure 2:**
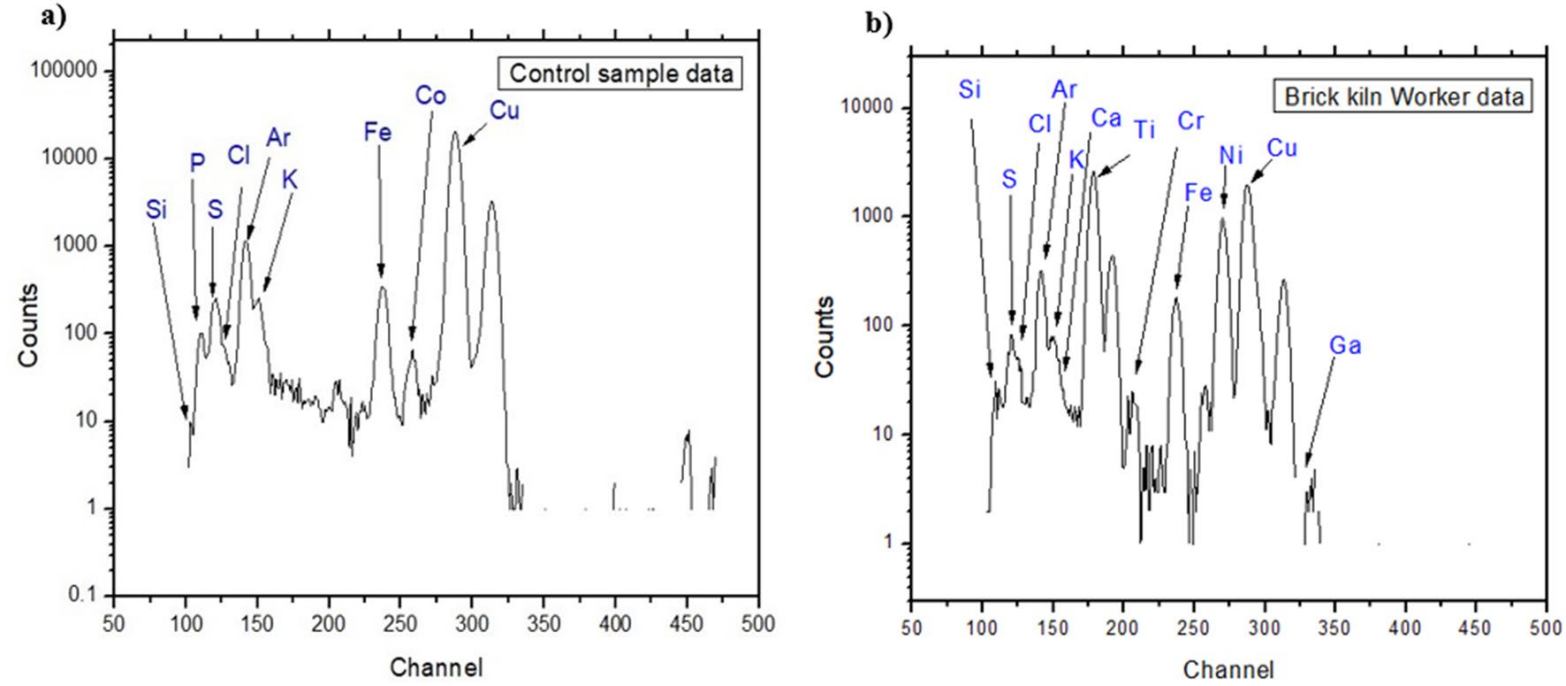
PIXE analysis of blood samples from (**a**) control subjects and brick kiln occupants (**b**) using 3 MeV proton energy at the National Centre for Physics, Islamabad, using a 5 MV tandem Accelerator. Note the difference between the peaks of certain heavy metals that are absent in control data. The concentration of titanium (Ti), chromium (Cr), nickel (Ni), and gallium (Ga) are most evident in brick kiln emission exposed group.

PIXE analysis showed that the elements detected included Si, P, S, Cl, K, Ca, Ti, Cr, Mn, Fe, Co, Ni, Cu, and Zn in the blood samples from brick kiln workers and control participants (supplementary Table 1). Chromium was only detected in the blood samples of brick kiln workers, whilst control participants had significantly higher levels of Co (*p* = 0.011), Mn (*p* = 0.017), Ni (*p* = 0.04), and Ti (*p* = 0.05). Although preparation and calibration were completed in such a manner to negate the effect of using 99.99% pure Cu strips as sample holders, Cu levels were significantly higher (*p* = 0.003) in the control group (11,883.1 ± 3701.3) than the brick kiln group (6296.7 ± 1966.0). No significant differences were observed between the blood samples retrieved from brick kiln workers and controls for Si (*p* = 0.408), P (*p* = 0.331), S (*p* = 0.518), Cl (*p* = 0.394), K (*p* = 0.596) and Ca (*p* = 0.334). The PIXE spectra for the elements detected is shown in Fig. 2.

#### Atomic absorption spectroscopy

Significantly higher levels of Cd (*p* = 0.000), Cr (*p* = 0.000), and Ni (*p* = 0.000) were detected in the blood of male workers compared to control participants. An increase in Zn levels was also found among the brick kiln group. Although, the results obtained from AAS and PIXE were comparable, PIXE were able to detect a wider profile of elements. Table 3 presents the metal concentration in whole blood obtained from this study as well from certified reference material previously used^46^ and provided by Seronorm^®^ whole blood trace element level 2 (Ref. 210205) reference material by Sero AS (Billingstade, Norway).

**Table 3:** Mean ± SEM heavy metals (lead, cadmium, nickel and chromium) concentration in whole blood of control and brick kiln male workers as well as from Seronorm certified reference material.

Heavy metals	Control	Workers	*P* value (correlation)	Certified reference material (µg/L)*
Cd (µg/dl)	2.37 ± 0.01	5.14 ± 0.02***	*P* = 0.000	0.565
Ni (µg/dl)	3.88 ± 0.01	6.40 ± 0.02***	*P* = 0.000	1.81
Zn (µg/dl)	1.24 ± 0.01	1.10 ± 0.01	*P* = 0.274	650
Cr (µg/dl)	2.02 ± 0.01	5.27 ± 0.02***	*P* = 0.000	1.18

Seronorm^®^ whole blood trace element level 2 (Ref. 210205) reference material by Sero AS (Billingstade, Norway)^46^.

Values are expressed as mean ± SEM. *, **, *** indicates significant difference at probability *p* < 0.05, *p* < 0.01 and *p* < 0.001 compared to control.

#### Body mass index

A significant decrease (*p* < 0.001) in BMI of workers was observed compared to unexposed men. Among the control samples, the average BMI was 26.9 ± 0.77, while those in brick kiln workers was 23.0 ± 0.23 (Table 4).

**Table 4:** The effects of heavy metal burden on BMI, blood parameters, biochemical profile, and reproductive hormones concentration of brick kiln male workers and controls.

Blood parameters	Control	Exposed	*P* value statistics
Average BMI (Kg/m^2^)	26.9 ± 0.77	23.0 ± 0.23***	*P* = 0.000
WBC (1 × 10^3^)	8.17 ± 0.40	8.84 ± 0.25	*P* = 0.163
HGB (g/dL)	14.1 ± 0.26	12.5 ± 0.41***	*P* = 0.001
RBC (1 × 10^3^)	5081.8 ± 63.7	4295.4 ± 89.2***	*P* = 0.000
HCT (%)	43.7 ± 0.84	38.7 ± 1.12***	*P* = 0.001
MCV (fL)	87.5 ± 0.92	82.5 ± 1.52**	*P* = 0.007
MCH (pg)	28.4 ± 0.36	26.5 ± 0.63*	*P* = 0.012
MCHC (g/dL)	32.5 ± 0.18	32.0 ± 0.25*	*P* = 0.088
RDW-CV (%)	13.6 ± 0.17	15.8 ± 0.49***	*P* = 0.001
RWD-SD (fL)	42.2 ± 0.46	46.3 ± 0.95***	*P* = 0.001
PLT (1 × 10^3^)	246.9 ± 11.6	293.1 ± 12.95**	*P* = 0.010
MPV (fL)	8.99 ± 0.12	9.02 ± 0.12	*P* = 0.898
PDW (%)	15.4 ± 0.04	15.5 ± 0.03	*P* = 0.194
PCT (%)	0.22 ± 0.01	0.260 ± 0.01*	*P* = 0.029
**Oxidants/antioxidants concentrations**			
SOD (U/min)	16.9 ± 0.72	14.4 ± 0.49**	*P* = 0.006
POD (nmole)	21.7 ± 2.22	19.2 ± 1.44	*P* = 0.393
ROS (μmol/min)	1.17 ± 0.06	1.50 ± 0.07***	*P* = 0.001
MDA (nmol /ml)	4.69 ± 0.36	5.28 ± 0.40*	*P* = 0.279
Protein estimation (g/dl)	11.6	8.95*	*P* = 0.013
**Reproductive hormones levels**			
FSH (mIU/ml)	12.19 ± 0.21	17.9 ± 0.44***	*P* = 0.000
LH (mIU/ml)	7.86 ± 0.12	10.8 ± 0.19***	*P* = 0.000
Testosterone (ng/ml)	5.80 ± 0.06	2.13 ± 0.12***	*P* = 0.000
Cortisol (µg/dl)	18.3 ± 0.41	30.4 ± 0.52***	*P* = 0.000

Values are expressed as mean ± SEM. *, **, *** indicates significant difference at probability *p* < 0.05, *p* < 0.01 and *p* < 0.001 compared to control.

### Blood parameters

Blood parameters are summarized in Table 4. Workers had significantly (*p* < 0.001) lower hemoglobin (HGB) levels, significantly (*p* = 0.001) lower red blood cells (RBC) levels, and significantly lower (*p* = 0.001) total haematocrit (HCT) concentrations than control samples. Mean corpuscular volume (MCV) (*p* = 0.007), mean corpuscular hemoglobin (MCH) (*p* = 0.012), and mean corpuscular hemoglobin concentration (MCHC) levels (*p* = 0.088) were also significantly lower in the worker group. In comparison, red cell distribution width coefficient of variation (RDW-CV) levels (*p* < 0.001), red cell distribution width standard deviation (RWD-SD) (*p* = 0.001), plateletcrit (PCT) (*p* = 0.029), and platelets count (PLT) (*p* = 0.01) were significantly higher in worker men compared to the control group. There was, however, no comparable change in mean platelet volume (MPV) (*p* = 0.898) and platelet distribution width (PDW) (*p* = 0.194) between the groups.

### Biochemical analysis

The biochemical studies showed a significant decrease (*p* = 0.006) in levels of SOD from 16.9 ± 0.72 U/min in the control group to 14.4 ± 0.49 U/min in the worker group (Table 4). A highly significant increase in the levels of ROS (*p* = 0.001) was also evident in the worker group compared to the control group. The blood protein content was also significantly reduced (*p* = 0.013) in workers compared to the control group. No significant differences were observed for POD and MDA levels between the groups.

### Hormonal analysis

FSH levels were significantly increased (*p* = 0.000) in the worker group (17.9 ± 0.44mIU/ml) compared to the control group (12.1 ± 0.21mIU/ml). Workers also had significantly (*p* < 0.001) higher LH (10.8 ± 0.19 mIU/ml vs 7.86 ± 0.12 mIU/ml in control) and cortisol (*p* < 0.001) levels (30.4 ± 0.52 vs 18.3 ± 0.41 in control). Testosterone levels were however significantly (p = 0.000) lower in workers compared to control participants (2.13 ± 0.12 ng/ml vs 5.80 ± 0.06 ng/ml) (Table 4).

### Correlation of reproductive hormones LH, FSH, T and cortisol

**Figure 3:**
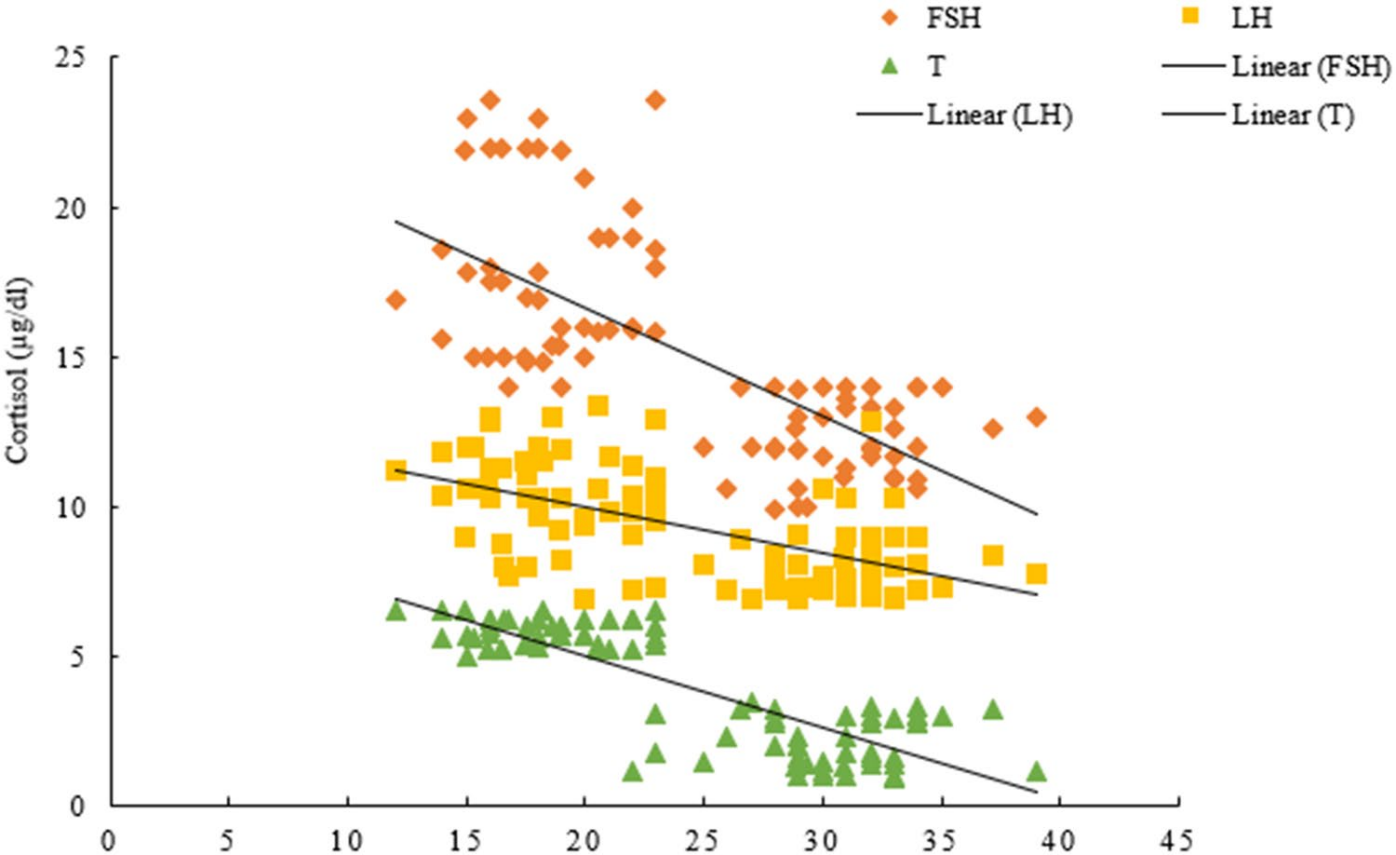
Plasma LH, FSH and testosterone concentration as a function of cortisol at brick kiln sites in adult male workers.

Pearson’s correlation showed that blood plasma cortisol levels had a significant negative correlation with LH (r =  − 0.580, *p* = 0.000), FSH (r =  − 0.676, *p* = 0.000), and T (r =  − 0.832, *p* = 0.000) concentrations (Fig. 3, supplementary Table 2). Further correlation analysis revealed a significant positive correlation between FSH and LH concentrations (r = 0.675, *p* = 0.000) as well as among FSH and testosterone (r = 0.749, *p* = 0.000) concentrations. Moreover, a significant positive correlation was evident between plasma concentrations of LH and testosterone (r = 0.623, *p* = 0.000).

### Correlation analysis between reproductive hormones and years of living at brick kiln sites

Table 5 summarizes the correlation analysis between reproductive and stress hormone concentrations and number of years living at brick kiln sites. A significantly negative correlation was observed with increasing number of years of living at brick kiln sites and plasma concentration of gonadotropins LH (r =  − 0.364, *p* = 0.014) and FSH (r =  − 0.777, *p* = 0.000), while a significant positive correlation with cortisol levels in blood plasma (r =  − 0.902, *p* = 0.000) was evident. The correlation of years of living at brick kiln sites with testosterone level showed no significant correlation (r = 0.136, *p* = 0.374). Cortisol levels showed a significant negative correlation with FSH (r =  − 0.946, *p* = 0.000) and LH (r =  − 0.380, *p* = 0.01). The levels of testosterone with FSH (r =  − 0.177, *p* = 0.246) and LH (r =  − 0.220, *p* = 0.146) also showed a significant negative correlation.

**Table 5:** Summary of the Pearson’s correlations between plasma LH, FSH, testosterone, and cortisol with number of living years at brick kiln sites.

Parameters	Correlation				
	Year of living (years)	Cortisol (µg/dl)	FSH (mIU/ml)	LH (mIU/ml)	Testosterone (ng/ml)
Year of living (years)	1				
Cortisol (µg/dl)	r = 0.902**	1			
	*P* = 0.000				
FSH (mIU/ml)	r = − 0.777**	r = − 0.946**	1		
	*P* = 0.000	*P* = 0.000			
LH (mIU/ml)	r = − 0.364*	r = − 0.380*	r = 0.335*	1	
	*P* = 0.014	*P* = 0.01	*P* = 0.024		
T (ng/ml)	r = 0.136	r = 0.164	r = − 0.177	r = − 0.22	1
	*P* = 0.374	*P* = 0.283	*P* = 0.246	*P* = 0.146	

Pearson’s correlation and significant relation; Pearson’s correlation is shown with r whenever significant by *P*-value. ** Correlation is significant at the 0.01 level (2-tailed).

### Multiple variant analysis with principal component analysis

**Figure 4:**
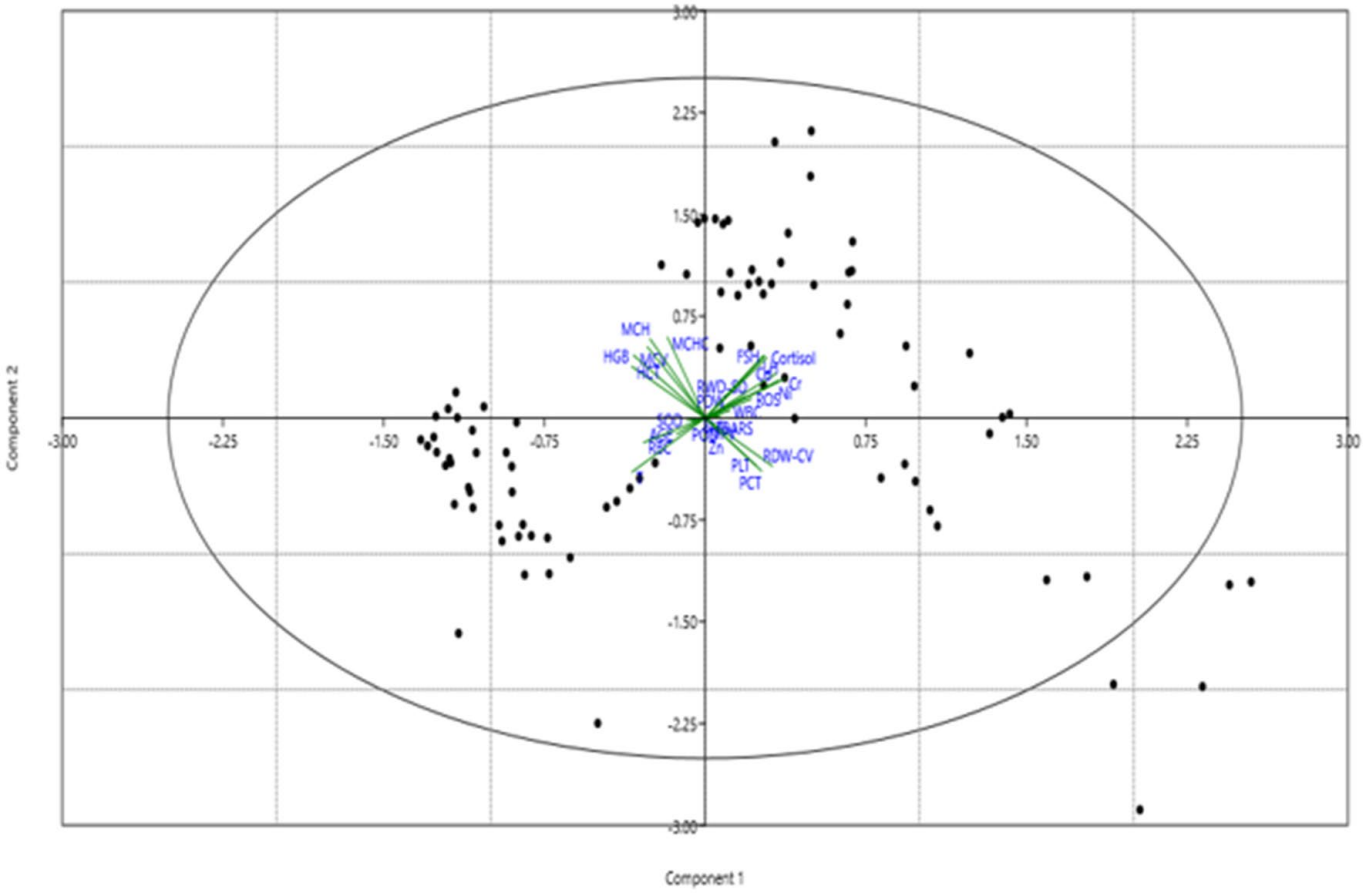
Scatter plot of principal component analysis (PCA) Component 1 versus PCA Component 2 scores. Each point is represented by a symbol denoting its analytical cluster and a line connecting it to the cluster centroid.

Components 1 and 2 both showed a maximum variation in the data, with most of the variation explained by component 1. Figure 4 shows the PCA scatter plot of component 1 versus PCA component 2 scores. The first six components in our data set explained 68.4% of the variation in the data, therefore, these components were considered (Table 6). The tested independent variables such as metals and toxic biochemicals such as Cd, Ni, Cr, ROS, hormones, and blood variables (RDW-CV, RDW-SD, PCT, PLT) are well explained in component 1. The variables Ni, Cr, Cd and ROS showed large positive loadings on component 1 and therefore, are important for other biochemical variables. Other variables that depicted strong positive loading on component 1 included RDW-CV, RDW-SD, PCT, PLT, LH, FSH and cortisol. There were strong negative loadings on component 1 for variables such as age, HGB, RBC, HCT, MCV, MCH, MCHC, and testosterone. The dependent and independent variables for the brick kiln worker group were closely placed towards the central axis, but with no significant correlation observed among control and brick kiln workers. Individuals from both the study groups were clattered with regard to component 1, showing that most of the tested variables were associated and better explained under component 1. The scree plot with the given eigenvalue also shows that maximum variation has been shown in component 1 (supplementary Fig. 3). Component 2 also showed strong positive plotting of Cr, Ni and Cd, with negative loading of Zn, RDW-CV, PLT, PCT and Testosterone. As component 1 presents the largest positive association of Cd, Ni, Cr, ROS, and cortisol, long-term exposure to these variables in brick kiln workers as compared to control group might raise public health concerns. All these variables act negatively to affect the blood variables (HGB, RBC, HCT, MCV, MCH, MCHC) that has also been shown through component 1. Another positive correlation observed in component 1 is for variables of reproductive hormones, LH and FSH that might show the reproductive potential of brick kiln workers to be at the risk for development of fertility problems. As component 2 showed the largest negative association with only the testosterone variable, it measures the history of HPG activity of brick kiln workers and thus, their reproductive potential.

**Table 6:** The table orders the eigenvalues from the larger to small.

PC	Eigenvalue	% variance
1	7.86	29.1
2	4.23	15.6
3	2.14	7.95
4	1.51	5.6
5	1.48	5.49
6	1.26	4.66
7	1.11	4.12
8	1.08	4.01
9	0.922	3.41
10	0.838	3.10
11	0.715	2.65
12	0.653	2.42
13	0.578	2.14
14	0.521	1.93
15	0.430	1.59
16	0.394	1.45
17	0.288	1.06
18	0.261	0.968
19	0.210	0.778
20	0.142	0.525
21	0.120	0.447
22	0.096	0.357
23	0.081	0.300
24	0.033	0.125
25	0.002	0.008
26	0.001	0.005
27	0.000	0.0018

*PC Principal component.

(This shows an ideal pattern as a steep curve, followed by a curve bend and then going to a straight line. The first three components have eigenvalues greater than 2 that explain > 50% of the variation in the data. These first three components are not adequate to explain the amount of variation in the data, so we took the first 8 components that have an eigenvalue greater than 1 as it explains approximately 80% of the variation in the data that is adequate to understand the variation in data).

## Discussion

The present investigation report on the demographic trends, intrinsic metal profile, and various health parameters of brick kiln workers at Rawalpindi, Punjab. This is a novel report, combining a unique set of techniques and approaches to characterize a brick kiln community, specifically males. Our results showed that 84% of the kiln workers were illiterate; this is supported by recent study conducted in India where illiteracy rate found at brick kiln sites was 56%^47^. Some of the individuals who participated in the present study were also addicted to various drugs such as smoking (22%). Previous studies have also reported that smoking trend is quite common in brick kiln workers^26^.

The findings further indicate that workers may not have access to health and welfare facilities since workers presented with a multiple of health issues including allergies, kidney, stomach as well as pulmonary disorders. Likewise, findings of Patil reported the prevalence of similar conditions at brick kiln sites present at village Karad Taluka, India. On a global scale, occupational risk factors cause respiratory illness that accounts for 13% of chronic obstructive pulmonary disease and 11% of asthma^25^. The development of respiratory diseases, multiple musculoskeletal symptoms, cancers and other health impacts that give rise to grave public health concerns among workers may be triggered by some demographic variables mentioned earlier in the text, i.e. prolonged/direct exposure to drugs via smoking, or exposure to brick kiln pollutants such as heavy metals^23,25,48,49^.

To screen exposure to heavy metals as possible triggers of the health conditions detected, blood elemental screening was performed utilizing a combination of robust elemental analysis techniques. The elements detected included Si, P, S, Cl, K, Ca, Ti, Mn, Fe, Co, Ni, Cu, and Zn. Determination of heavy metals in whole blood showed a remarkable increase in levels of Cd, which is known to induce reproductive toxicity among males through multiple mechanisms^50^. An earlier study conducted by Jahan et al. (2016) showed similar results, where significant increases in Cd levels were observed in the blood of brick kiln workers compared to control samples. Recent studies have also found high levels of Cd in the blood of brick kiln workers^51^. Cadmium is accumulated in the liver and kidney tissues and can accumulate for years in the body^16^, with prolonged exposure resulting in edema, cell death and tubular destruction, that ultimately leads to endocrine disruption as well as musculoskeletal and cardiovascular diseases^15^.

Our results further revealed elevated levels of Cr detected through AAS in workers, which acts as a carcinogenic agent in humans^52^. Occupational exposure to Cr and its compounds may affect 300,000 workers annually and is a major concern for causing Cr-related diseases in industrial workers^19^. Previous work has also shown elevated levels of Cr in the blood of brick kiln workers that is considered to be associated with multiple health effects, including lung cancer, an altered immune response, and allergic reactions^53–55^. Chromium is also known to induce cellular toxicity via the production of ROS with subsequent cellular damage^56^. Elevated levels of nickel were also found in the blood of brick kiln participants. Previous findings suggest that Ni levels in blood were quite high in occupationally exposed individuals, which may lead to multiple health problems among males and females^57^, including cancers, dermatitis respiratory disorders, neurotoxicity, epigenetic changes, and reproductive diseases triggered via various mechanisms^58,59^. No significant differences were detected between the control and brick kiln samples for essential (P, S, Cl, K, Ca) and non-essential (Si) elements.

Interestingly, no chromium was detected in the blood of control participants although control participants had significantly higher levels of Cu, Co, Mn, Ni, and Ti, which may be introduced via other sources of anthropogenic or natural pollution. Elevated Co levels in blood serum are known to be associated with erythrocytosis, poses a risk to the development of heart and thyroid gland pathologies, and is responsible for occupational asthma and dermatitis^60^. Although manganese and Fe are naturally occurring metals which usually coexist in the environment such as in ground water, elevated levels in blood have been linked to metabolic diseases such as cardiovascular problems and atherosclerosis^61,62^. Similarly, even minute amounts of Ti in the body may exert toxic effects through altering cell cycle events and constriction of nuclear membranes that may lead to cell death^63^. In sum, the metals detected collectively induce multiple metabolic as well as reproductive disorders^20–23^, including various disorders of the heart, liver, skin, brain, kidney, liver, and respiratory disorders^64^. It can hence be concluded that the presence of elevated levels of hazardous metals in blood serum may have detrimental effects on the health of the brick kiln workers and participants living near the brick kilns.

A significant decreased BMI of kiln workers compared to control subjects was observed, with average BMI falling within the normal range. Of the brick kiln workers who participated in the present study, 10% were underweight. The low BMI values of workers may indicate poor worker health status and a compromised immune system, making them more prone to various allergies, musculoskeletal problems, respiratory disorders, and viral diseases^41^, whilst various other socio-economic and occupational factors such as physical labor may also explain this finding. Environmental exposure to metals such as cadmium may also contribute to a negative correlation with BMI as previously suggested^65^. Other findings similarly suggest that exposure to even low levels of Cd, may impart significant negative effects on body weight^66^. Recent literature however suggests that there is not a single metal directly associated with BMI, although altered BMI is indirectly linked to metals that are known to act as endocrine disruptors, induce oxidative stress, and affect glucose metabolism^67^. Our data is in contrast with results of^41^ and supported by previous work of Kamal et al.^2^, where average BMI in control subjects was greater than the BMI of brick kiln workers. Furthermore, our results showed that overweight (18%) and obese (10%) brick kiln workers were present in our study. A recent study also reported an increased number of overweight (50%) and obese kiln workers (35.7%)^26^.

The findings of blood parameters showed a decrease in RBC and HGB levels that might be linked to the presence of heavy metals emitted from the brick kiln environment and detected in the blood of workers. Previous findings suggested a similar decrease in RBC levels in blood of brick kiln workers as compared to the control group. As RBC is produced by the hematopoietic tissues of the kidney/spleen, studies have confirmed that internal bleeding from a damaged kidney due to heavy metal exposure may result in the decreased production of RBC^68^. Our findings are further supported by previous work of Fazio et al., (2014) in which lowered RBC and WBC levels were evident among fish exposed to metal pollution^69^. Chromium (VI) is known to induce cellular toxicity in blood lymphocytes via the production of ROS that causes cellular damage that may affect the production of red and white blood cells^56^. Another study further showed that in vitro incubation of erythrocytes and lymphocytes with different concentrations of potassium chromate resulted in a dose-dependent increase in reactive oxygen species along with a reduction in the antioxidant capacity of cells^70^. Kamal et al.^2^ have also suggested that a variety of brick kiln emitted particles may induce cellular toxicity in actively dividing cells (e.g. bone marrow cells). In our study, a decrease in haematocrit, MCV, MCH, and MCHC levels were also noted among brick kiln workers that might be due to the toxic effects of heavy metals on cell ROS metabolism.

Antioxidant enzymes including catalase and peroxidases play an important role in reducing the threatening effects of heavy metals. Turkez et al.^71^ have reported that exposure to heavy metals elevates oxidant levels and reduces antioxidant concentrations. A noteworthy decrease in SOD and POD levels were observed in the worker group, which might be due to rising levels of heavy metals in the blood of workers. Previous findings also reported a decrease in SOD level in brick kiln workers as compared to the control group. The findings of the current study are supported by previous reports of Jahan et al.^51^ and David et al.^72^, in which brick kiln emissions were shown to decrease the levels of antioxidant enzymes in brick kiln workers^20,]^.

Studies suggest that transition metals such as cadmium, nickel, and chromium act as catalysts in the oxidative reactions of biological macromolecules^73^;^74^. Our study shows that the brick kiln worker group had a significantly increased number of ROS and MDA detected in blood as compared to control participants. Similar results have been previously reported, where increased levels of MDA were evident among brick kiln workers as compared to controls^12,51^. High levels of ROS disturbs the body’s normal metal ion homeostasis causing oxidative stress, which leads to suppression of the body’s own antioxidant protection, resulting in lipid peroxidation and protein modification^72^. Increased oxidative stress may result in chromium tetravalent induced cellular damage as suggested by previous studies^56,70^. However, further studies are required to understand the effect on antioxidant enzyme and ROS concentrations following heavy metal exposure at the molecular and cellular level.

For reproductive hormones, an increase in FSH and LH levels among brick kiln workers was found. Long term exposure to heavy metals may result in the deposition and burdening of heavy metals in the blood and other tissues, that may affect the plasma concentration of LH and FSH as suggested by one of our previous findings where female workers exposed to brick kiln emissions experienced Cd, Cr and Ni burden in blood^20^. Our findings are further supported by the work of Lafuente et al.^75^ in which rats exposed to cadmium chloride in drinking water experienced a decrease in LH and an increase in FSH concentrations. In response to high levels of LH, a corresponding increase in testosterone levels was expected which was confirmed by a positive correlation between LH and testosterone concentrations. However, decreased levels of testosterone in the blood plasma of brick kiln workers were noted compared to controls, which might be due to the presence of a high concentration of heavy metals in the blood of workers that increased oxidative stress associated with reproductive dysfunction.

It has been reported in previous studies that many components of the hypothalamic-pituitary–gonadal axis are downregulated by plasma glucocorticoids such as cortisol. These effects are mediated either at the hypothalamus and pituitary level or by actions on the responsiveness of target tissues to gonadal hormones^76,77^. The present study reported an increase in the concentration of cortisol among brick kiln workers. The findings of the current research further report a significant negative correlation of cortisol concentrations with pituitary gonadotropins LH and FSH as well as testosterone in blood plasma. In humans, exposure to cortisol causes a significant decrease in testosterone production^78^. Many other studies have also reported a negative correlation of cortisol hormone with sex steroids^79–82^. The increase in cortisol is thought to be responsible for a decrease in sex steroid production from Leydig cells and therefore, can be considered accountable for reproductive function in men.

Based on the current findings, it is evident that brick kiln workers are at the verge for developing public and reproductive health concerns. It is recommended that there must be recurrent inspection of the monitoring strategies for brick kiln emissions as well as regular medical checks of workers to ensure an improved quality of life among laborers^20,45^. Governmental bodies should also monitor issues related to bonded labor and social risks associated with the brick kiln industry^3^. Although we conducted the present study choosing one brick kiln area (District Rawalpindi), future studies should focus on the assessment of data on a national scale to understand the prevalence of social and public health concerns at the occupational setup. There is also a need to conduct similar studies concentrated on worker health and reproductive health to advise the availability of proper health facilities among occupational workers exposed to a variety of kiln emitted pollutants. Furthermore, similar studies should be planned at the molecular level to assist in explaining the exact impact of heavy metals on biological variables, whilst the geochemical profile of brick kiln starting materials and bricks should also be investigated as a potential direct route of exposure to heavy metals.

## Conclusion

In conclusion, this study showed the viability of external beam PIXE to provide elemental data on blood samples serving as biomarkers for exposure to toxic elements. The average values of Co, Ni, Ti, Cr, Mn and Fe were found to be higher than permissible limits recommended by the FDA. The study further summarized that the heavy metal burden detected in the blood of workers is thought to be responsible for the reduction in BMI, alteration in levels of blood parameters, decrease in antioxidant levels, and increased oxidant production. The heavy metal burden in blood further increased the stress response, resulting in elevated production of cortisol, which ultimately affected the HPG axis by altering the production of gonadotropins (LH, FSH). Reduction in the production of sex steroid testosterone was evident. Therefore, it is believed that due to poor socio-economic conditions of workers and compromised biochemical and reproductive status, these young adult men are on the verge of developing reproductive problems. Future studies should also include geochemical properties of bricks and brick kiln making materials as contributory factors to the heavy metal profile of blood sampled from brick kiln workers. Because of the negligence of health concerns of brick kiln workers, there is a need to plan further studies to report the public health and reproductive health issues faced by local communities of brick kiln workers, so that monitoring strategies for brick kiln emissions and provision of medical checks of workers can be ensured to improve the quality of life among laborers.

## Supplementary Information

Supplementary Information.

## Data Availability

All data generated or analyzed during this study are included in this published article and its supplementary information files.
