# Supplementary material for: Biochemical and reproductive biomarker analysis to study the consequences of heavy metal burden on health profile of male brick kiln workers
Source: Sci Rep. 2022 May 3;12:7172. doi: 10.1038/s41598-022-11304-7 (PMC9065070; doi:10.1038/s41598-022-11304-7)
Supplement: Supplementary file 1 — Supplementary Information. [file 41598_2022_11304_MOESM1_ESM.pdf]

Biochemical and reproductive biomarker analysis to study the consequences of heavy metal burden on health profile of male brick kiln workers

Mehwish David[1], Sarwat Jahan[1], Javaid Hussain[2], Humaira Rehman[3], Karen J Cloete[4,5] Tayyaba Afsar[6], Ali Almajwal[6], Nawaf W. Alruwaili[6,] Suhail Razak[6*]

SUPPLEMENTARY MATERIAL

Supplementary Table 1. Elemental levels detected in blood samples from the test and control group.

Metals µg/mL	Control	Workers	P-value statistics
**Silicon (Si)**	1323.65±238.96	1183.63±222.33	p=0.408
**Phosphorus (P)**	592.70±143.47	608.38±83.6	p=0.331
**Sulphur (S)**	709.94±225.27	786.01±213.31	p=0.518
**Chlorine (Cl)**	396.87±111.75	556.15±120.21	p=0.394
**Potassium (K)**	471.53±161.19	495.72±96.88	p=0.596
**Calcium (Ca)**	94.33±35.93	92.33±65.50	p=0.334
**Titanium (Ti)**	3271.80±3152.50	1345.58±634.8	p=0.052
**Chromium (Cr)**	--	23.9±4.01	p=0.000
**Manganese (Mn)**	979.24±577.92	64.44±39.42	p=0.017
**Iron (Fe)**	138.22±66.46	144.05±20.52	p=0.055
**Cobalt (Co)**	2243.26±1381.42	209.41±105.47	p=0.011
**Nickel (Ni)**	1818.41±974.09	1212.98±147.40	p=0.045
**Copper (Cu)**	11883.11±3701.31	6296.72±1966.0	p=0.003
**Zinc (Zn)**	225.94±18.73	179.10±33.92	p=0.580

Silicon = Si, Phosphorus = P, Sulphur = S, Chlorine = Cl, Potassium = K, Calcium = Ca, Titanium = Ti, Chromium = Cr, Manganese = Mn, Iron= Fe, Cobalt = Co, Nickel = Ni, Copper = Cu, Zinc = Zn

Supplementary Table 2. Summary of the Pearson's correlations between plasma LH, FSH, testosterone and Cortisol in male workers and control

**Parameters**	**Correlation**			
	**Cortisol (µg/dl)**	**FSH (mIU/ml)**	**LH (mIU/ml)**	**T (ng/ml)**
**Cortisol (µg/dl)**	r=1			
**FSH (mIU/ml)**	r=-.676**<br>p=0.000	r=1		
**LH (mIU/ml)**	r=-.580**<br>p=0.000	r=0.675**<br>p=0.000	r=1	
**T (ng/ml)**	r=-.832**<br>p=0.000	r=0.749**<br>p=0.000	r=.623**<br>p=0.000	r=1

Pearson's correlation and sets significant relation; Pearson's correlation is shown with r whenever significant by P -value. ** Correlation is significant at the 0.01 level (2-tailed)

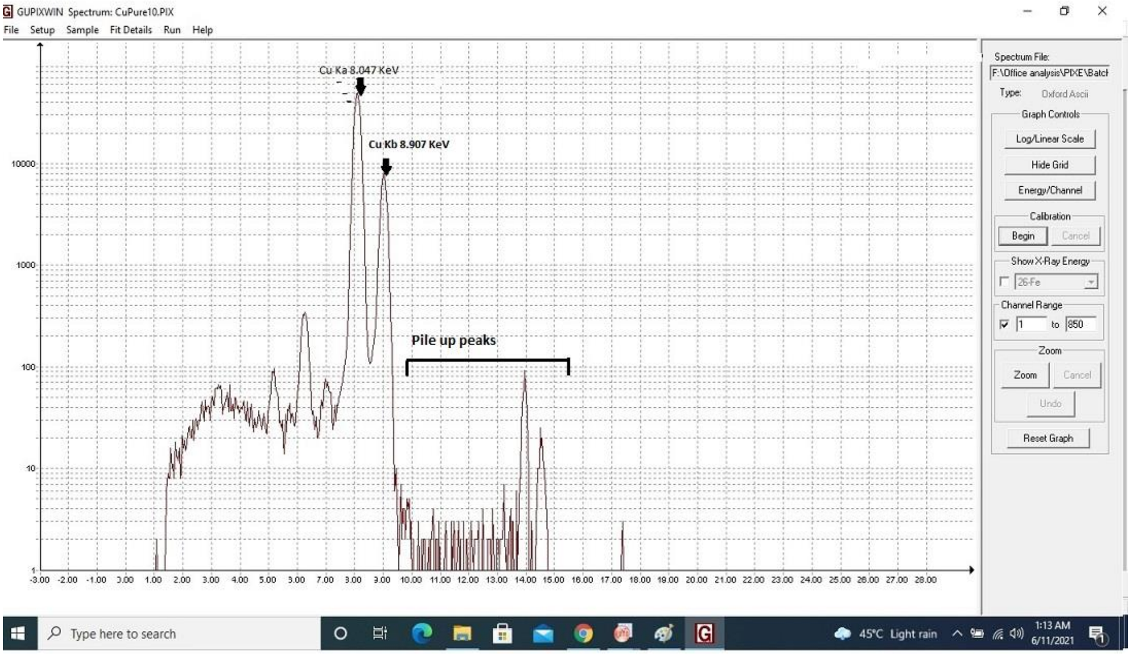

Supplementary Fig 1.0 GUPIX PIXE System was calibrated using pure Cu standard. Channel to energy conversion at 285/Cu Ka: 8.047keV and 311/Cu Kb: 8.905 keV was performed to calibrate the PIXE setup. H- value was fixed to 0.001531 Str. Si-Escape peak was found at channel no/energy 234/6.4 keV.

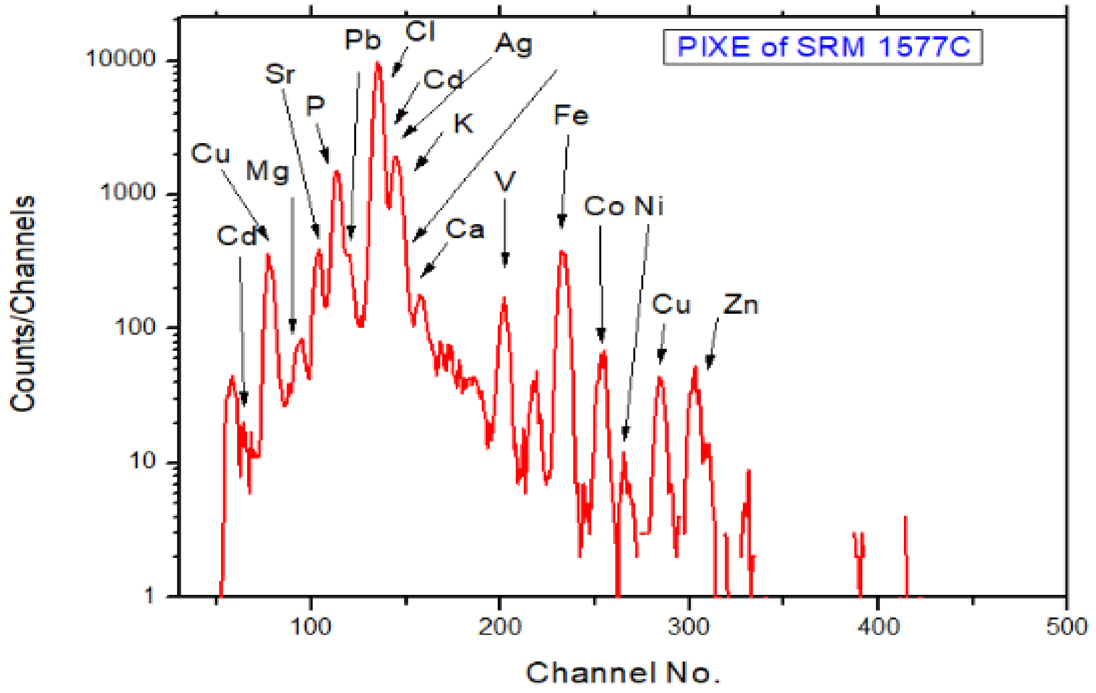

Supplementary Fig 2. PIXE analysis of SRM 1577c of bovine liver using 3MeV proton energy at NCP, Islamabad using 5 MV tandem Accelerator.

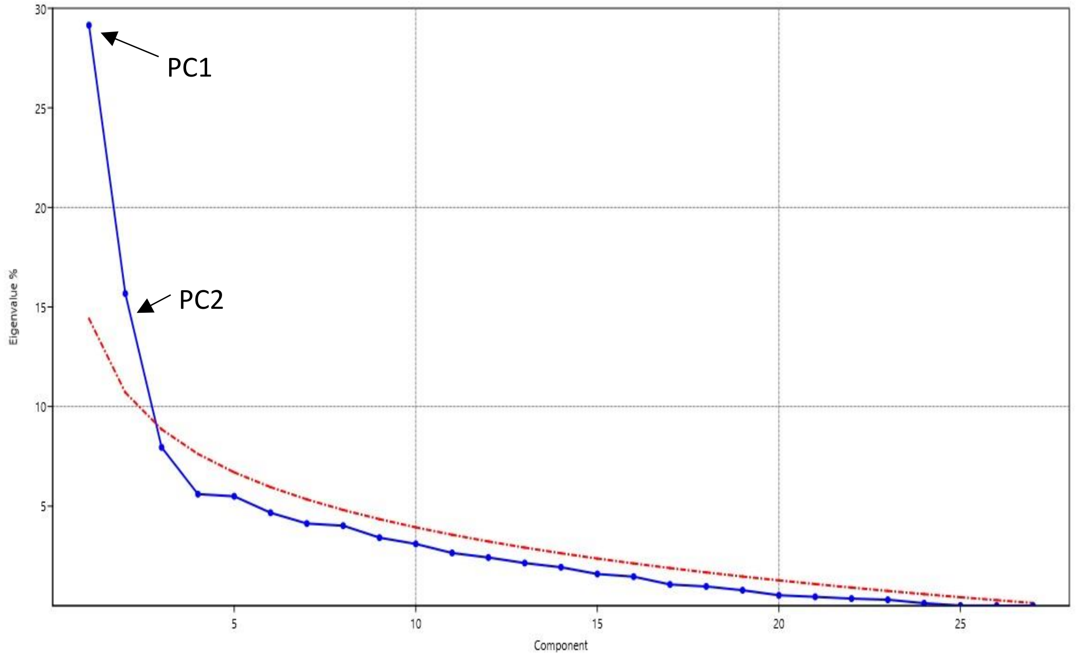

Supplementary Fig 3. A scree plot where it orders the eigenvalues from the largest to the smallest. This shows an ideal pattern is a steep curve, followed by a curve bend and then going to be straight line. In our results first, three components have eigenvalues greater than 2 that explain > 50% of the variation in the data. The scree plot indicates that the eigenvalues start to

form a bend and then a straight line after the fourth component. These first three components are not adequate to explain the amount of variation in the data, so we took first 8 components that have eigenvalues greater than 1, and it explains approximately 80% of the variation in the data that is adequate to understand the variation in data
